# Characterization of Bee Pollen: Physico-Chemical Properties, Headspace Composition and FTIR Spectral Profiles

**DOI:** 10.3390/foods10092103

**Published:** 2021-09-06

**Authors:** Saša Prđun, Lidija Svečnjak, Mato Valentić, Zvonimir Marijanović, Igor Jerković

**Affiliations:** 1 Department of Fisheries, Apiculture, Wildlife Management and Special Zoology, Faculty of Agriculture, University of Zagreb, Svetošimunska cesta 25, 10000 Zagreb, Croatia; sprdjun@agr.hr (S.P.); valentic.mato@gmail.com (M.V.); 2 Department of Food Technology and Biotechnology, Faculty of Chemistry and Technology, University of Split, Ruđera Boškovića 35, 21000 Split, Croatia; zmarijanovic@ktf-split.hr; 3 Department of Organic Chemistry, Faculty of Chemistry and Technology, University of Split, Ruđera Boškovića 35, 21000 Split, Croatia; igor@ktf-split.hr

**Keywords:** bee pollen, physico-chemical properties, headspace composition, FTIR spectral profiles

## Abstract

Chemical characterization of bee pollen is of great importance for its quality estimation. Multifloral and unifloral bee pollen samples collected from continental, mountain and Adriatic regions of Croatia were analyzed by means of physico-chemical, chromatographic (GC-MS), and spectroscopic (FTIR-ATR) analytical tools, aiming to conduct a comprehensive characterization of bee pollen. The most distinctive unifloral bee pollen with regard to nutritional value was *Aesculus hippocastanum* (27.26% of proteins), *Quercus* spp. (52.58% of total sugars), *Taraxacum* *officinale* (19.04% of total lipids), and *Prunus* *avium* (3.81% of ash). No statistically significant differences between multifloral and unifloral bee pollen from different regions were found for most of the physico-chemical measurement data, with an exception of melezitose (*p* = 0.04). Remarkable differences were found among the bee pollen HS VOCs. The major ones were lower aliphatic compounds, monoterpenes (mainly linalool derivatives, especially in *Prunus* *mahaleb* and *P.* *avium* bee pollen), and benzene derivatives (mainly benzaldehyde in *T.* *officinale* and *Salix* spp.). Aldehydes C_9_ to C_17_ were present in almost all samples. FTIR-ATR analysis revealed unique spectral profiles of analyzed bee pollen exhibiting its overall chemical composition arising from molecular vibrations related to major macromolecules—proteins, lipids, and carbohydrates (sugars).

## 1. Introduction

Honey bees (*Apis mellifera* L.) collect pollen from plant anthers, mix it with a small amount of the secretion from salivary glands or nectar (10%), and place it in specific baskets on their hind legs (*corbiculae*)—the pollen stored in this way is known as bee pollen or pollen load. Pollen grains, depending on the plant species, differ in shape, color, size and weight [1], which causes great variability in the composition and visual appearance of bee pollen. The chemical compositions of bee pollen have drawn worldwide research interest, covering broad aspects ranging from plant physiology to biochemistry [2]. More than 200 compounds have been found in the bee pollen of various botanical origin, including proteins (ca. 22.7%), essential amino acids (ca. 10.4%), carbohydrates (ca. 30.8%), lipids (ca. 5.1%) and fatty acids (ca. 0.4%), phenolic compounds (ca. 1.6%), enzymes and coenzymes, and vitamins and small amount of volatiles [3,4,5,6]. Bee pollen is one of the most complete and nutritious foods in nature as it contains almost all the essential nutrients for humans, which are not commonly found in foods of animal origin. High nutritional value of bee pollen originates from the content of essential substances such as carbohydrates, proteins, lipids, vitamins, and minerals. Bee pollen also contains important bioactive compounds, such as polyphenols and flavonoids [4,5,6,7,8,9] which are known antioxidants [10] responsible for various biological activities. Furthermore, bee pollen contains all the amino acids essential for human body [11] in concentrations that are five to seven times higher than those found in traditional high protein foods [4].

The unifloral bee pollen has characteristic organoleptic and biochemical properties similar to the pollen grains of the original plant species from which the pollen was collected, while multifloral bee pollen reflects diverse chemical properties originating from more than two plant species. Minor variations in the composition of bee pollen can be caused by differences in foraging area (primarily, availability of polleniferous plant species), environmental conditions including soil type [12], seasonal and regional conditions, beekeeping activities, and bee pollen harvesting method, but the major variations in the composition and nutritional value of bee pollen arise from various botanical origin. Pollen of different plant species differs significantly in the content of proteins, lipids, minerals and vitamins and consequently, bee pollen has a different nutritional value. The proportion of proteins can vary from only 2% to 61%, carbohydrates from 15% to 50%, and starch up to 18% [13]. Chemical characterization of bee pollen is of great importance for its quality estimation. Several countries, namely Argentina [14], Brazil [15], Bulgaria [16], Poland [17] and Switzerland [18], have established national quality standards for bee pollen intended for human consumption.

No mention of pollen odor appeared in the literature until the 1920s. Afterward, the pollen of different plant species was described with distinctive odors. Over the last two decades, the analyses of pollen headspace have confirmed that pollen possesses species-specific odors and that these are chemically distinct from the odors from other floral parts [19]. These findings gave new inputs to study the role of pollen odor in pollination ecology, especially in attracting flower-visiting insects and modulating their foraging behavior and in defending pollen against pollen-feeding organisms and pathogens [20]. The pollen odors originate from pollenkitt, a sticky, oily coating comprising lipids and pigments, which loosely covers the surface of pollen grains of many plants [21]. In comparison with total flower odor, the pollen odor was found to be chemically distinct among the species [22]. Three major classes of compounds were reported in pollen floral scents, namely fatty-acid derivatives, isoprenoids, and benzene derivatives [23].

Bee pollen has been studied for many years, primarily in terms of physico-chemical characterization by classical analytical methods. Fourier transform infrared-attenuated total reflectance (FTIR-ATR) spectroscopy has become an increasingly used analytical tool for the studying bee pollen in the last few years, i.e., since 2017 [11,24,25,26,27], due to numerous advantages. Moreover, FTIR-ATR spectroscopy is well known as a rapid, reliable, non-destructive, reagent-free, and easy-to-use analytical technique that provides a unique chemical fingerprint of analyzed samples, reflecting its overall chemical composition based on functional group vibrations. It was successfully employed for preliminary spectral analysis of bee pollen by Anjos et al. [11], who indicated FTIR-ATR spectroscopy as a useful technique for assessing bee pollen composition. Further investigations [24,25,26,27] confirmed its reliability for bee pollen chemical characterization by providing a detailed assignation of the molecular vibrations observed in FTIR-ATR spectra of various bee pollen types.

To the best of our knowledge, there is no systematic study on chemical characterization of different unifloral and multifloral bee pollen types gathered by honey bees in different climatic and geographical regions. Therefore, the aims of the present study were: (i) to obtain bee pollen collected by the honey bees from three different climatic-geographical regions in Croatia (continental, mountain, and Adriatic) during the spring; (ii) to separate and identify unifloral bee pollen; (iii) to investigate the bee pollen physico-chemical properties, headspace composition and FTIR profiles in order to provide a detailed insight in its composition and nutritional value.

## 2. Materials and Methods

### 2.1. Sampling of Bee Pollen

Bee pollen samples were collected using front-mounted pollen traps placed at the entrance of the Langstroth-Rooth hives situated at three experimental apiaries in continental region-CR (Krapina; 46°9′4.20″, 15°52′4.76″), mountain region-MR (Otočac; 44°48′46.78″, 15°21′35.58″), and Adriatic region-AR (Senj; 44°59′6.20″, 14°54′24.29″) of Croatia. Five honey bee experimental colonies were selected at each location. The collection of bee pollen was performed every 15 days, starting from 1 April to 15 June. After removal from the pollen traps, the bee pollen samples were stored in plastic bottles at −18 °C until the analyses. In total, 16 pooled (multifloral) bee pollen samples were collected. The pollen loads of the same botanical origin were isolated from the pooled samples using a soft painting brush (preliminary selection was based on the visual appearance/color, while further identification of the botanical origin relied upon the confirmation of uniflorality by melissopalynological analysis). In total, 48 unifloral subsamples were obtained from pooled samples. Both sample sets (multifloral and unifloral) were subjected to further analyses.

### 2.2. Determination of Botanical Origin-Melissopalynological Analysis

The slides for melissopalynological analysis were prepared according to the method of Barth et al. [27]. Identification and counting of pollen grains were performed according to Von der Ohe et al. [28] under a light microscope (Carl Zeiss Axio, Carl Zeiss, Jena, Germany) at a magnification of 400–1000× attached to a digital camera Axiocam 208 Color (Carl Zeiss, Jena, Germany), and coupled to an analysis software (ZEN 3.1 blue edition, Carl Zeiss, Jena, Germany). The identification of pollen grains was supported by using a collection of reference samples of pollen grains in the form of native preparations (internal collection of the Department of Fisheries, Beekeeping and Special Zoology, University of Zagreb Faculty of Agriculture), and literature data [29,30]. At least 500 pollen grains were counted in each slide. The sample that contained more than 80% [31] of pollen grains of particular plant species was considered as unifloral bee pollen.

### 2.3. Physico-Chemical Analyses

#### 2.3.1. Moisture Content (%)

The moisture content was determined by vacuum drying according to the AOAC 969.38 method [32]. In total, 3 g of the bee pollen sample was placed in a dried, cooled in desiccant, and weighed aluminum dish with a lid. The dish was covered with the appropriate lid and weighed. The lid was then removed and the aluminum dish and the lid were placed in a vacuum dryer (Vacuum dryer/Lyophilizer: Christ, model: Alpha LSC plus) with previously set parameters (temperature 60 ± 20 °C, pressure 6.7 kPa <50 mm Hg). After the appropriate drying time (9 h), the dish was covered with a lid, cooled for at least 45 min in a desiccator and weighed. The dish was returned to the dryer for 1 h, cooled and weighed. The procedure was repeated until a constant mass was reached (difference of two successive weighing ≤ 1 mg). The differences between two parallel determinations of the same sample did not exceed 0.3% of the determined value.

#### 2.3.2. Ash Content (%)

The ash content was determined gravimetrically, according to the AOAC 923.03 [33]. In total, 3 g of the bee pollen sample was placed in an annealed porcelain crucible (550 °C), cooled in a desiccator and weighed, and distributed evenly over the crucible. Using an electric heater, the sample was slowly heated until it carbonizes. When the smoke stopped developing, the crucible was transferred to a muffle furnace and preheated to 550 °C until ash was obtained. If the residue in the crucible contained unburned carbon particles, it was moistened with a small amount of distilled water and 2 mL of concentrated HNO_3_ (cover the ash) after cooling. The crucible covered with a watch glass was heated on an electric heater for about an hour. After the acid was evaporated, the content was dried and burned again in a muffle furnace. Upon completion of incineration, the crucible was placed in a desiccator to cool and weighed to the ±0.1 mg. Incineration was repeated until a constant mass.

#### 2.3.3. Protein Content (%)

The bee pollen sample was weighed (0.8 g ± 0.1 mg), wrapped and placed in a test tube. The 10 g of Na_2_SO_4_ and 0.1 g of CuSO_4_ were weighed and transferred to a test tube. In total, 15 mL of concentrated H_2_SO_4_ was added and the test tube was placed on an incinerator stand and left for 30 min. The tube was connected to a suction system and placed together in a preheated block (420 °C). The destruction lasted about 1 h. The sample was taken out and allowed to cool. Afterward, 75 mL of water was added. The test tube with burned sample and added water was placed in a tray of the distillation apparatus and a template (Erlenmeyer flask with 25 mL of 0.1 M HCl and a few drops of indicator) was placed such that the hose was immersed in the flask. In total, 50 mL of 35% NaOH was added to the burnt sample in the test tube. The volume was controlled with a calibration tube on the back of the device. In total, 100 mL of distillate was collected. The pH of the distillate was checked with indicator paper and the sample was triturated with 0.1 M NaOH. The protein content was determined using the Kjeldahl method (AOAC 2001.11) [34], and protein was calculated using the conversion factor of 5.6 (N × 5.6).

#### 2.3.4. Total Lipid Content (%)

The total lipid content was determined gravimetrically, according to the AOAC 963.15 [35]. In total, 3 g of the bee pollen sample was weighed and added into a 250 mL beaker, with a few boiling beads, 45 mL of boiling water and 55 mL of 8 M HCl. The glass was covered with a watch glass, heated on an electric heater, and after boiling, it was measured for 15 min. After cooling, the watch glass was washed with distilled water and the content was filtered through filter paper. The beaker was rinsed several times with water, and the contents of the filter paper were rinsed with water until negative to the chlorides with 0.1 M AgNO_3_. After digestion, the filter paper was transferred to a test tube, placed in a glass and dried for 2 h at 100 °C. After drying, the glass on which the extraction thimble was dried, the watch glass that covered the beaker and the beaker were wiped with a cotton ball soaked in petroleum ether and placed in the extraction thimble. This was followed by lipids extraction, according to Soxhlet. A Soxhlet flask with several boiling beads was dried at 100 °C (± 2 °C) for 1 h, cooled in a desiccator for 30 min and weighed on an analytical balance. The dried extraction thimble was placed in a Soxhlet apparatus extractor, connected with the flask and 150 mL of petroleum ether was added. The extraction lasted 4 h, i.e., the extractor was emptied at least 30 times. The solvent was distilled off, the residue was evaporated on a water bath and the flask was dried in an oven at 100 (± 2 °C). The flask was cooled in a desiccator for 30 min, weighed on an analytical balance and dried again for 30 min at a temperature of 100 °C (± 2 °C) to the constant weight. The total lipid content (%) was calculated from mass of extracted lipid (g) × 100/sample weight (g).

#### 2.3.5. Determination of Sugar Content (%) by HPLC Method

Sugar analysis of bee pollen samples was performed by HPLC (high performance liquid chromatography) (Shimadzu Corp., Kyoto, Japan) consisted of a refractive index detector (RID-10A, Shimadzu Corp., Kyoto, Japan), HPLC column (Agilent Zorbax NH2, 4.6 × 250 mm, particle size 5 μm, Santa Clara, CA, USA), quaternary pump (LC-20AD, Shimadzu Corp., Kyoto, Japan), oven (CRO-20AC, Shimadzu Corp., Kyoto, Japan) and autosampler (SIL-10AF, Shimadzu Corp., Kyoto, Japan). The mobile phase consisted of acetonitrile (J. T. Baker, Avantor, Gdansk, Poland), and ultra-purified H_2_O in a volume ratio of 75:25. Isocratic elution of mobile phase was performed with the flow rate of the mobile phase was 1.3 mL/min, the column temperature was 30 °C, and the injection volume was 10 µL. The total time of analysis was 30 min. HPLC grade standards of sugars were used for identification and quantification. Anhydrous glucose, fructose, sucrose, and melezitose hydrate were purchased from Sigma (St. Louis, MO, USA), xylose and maltose monohydrate from Kemika (Zagreb, Croatia), and raffinose pentahydrate from Fluka (Darmstadt, Germany). The identification was performed based on the comparison of retention time of individual separated sugar to corresponding pure component while the quantification was performed using external calibration method. This method was used to determine glucose, fructose, sucrose, maltose, melezitose and raffinose. The identification and quantification were performed using the software LabSolution Lite Version 5.52 (Shimadzu Corp., Kyoto, Japan). The total sugar content was obtained by calculation as a sum of values of determined individual sugars.

### 2.4. HS-SPME/GC-MS Analysis

The headspace (HS) volatiles were extracted by a manual SPME (solid phase microextraction) fiber with a layer of divinylbenzene/carboxene/polydimiethylsiloxane (DVB/CAR/PDMS) from Supelco Co (Bellefonte, PA, USA). The fiber was conditioned according to the manufacturer’s instructions. For HS-SPME, the pollen grains (1 g) were placed in 15 mL glass vial and hermetically sealed with PTFE/silicone septa. The closed vial was placed in a water bath at 60 °C during equilibration (15 min), and the extraction time of 45 min was applied for HS-SPME. After the sampling, the SPME fiber was withdrawn into the needle, removed from the vial, and inserted into the injector (250 °C) of the GC-MS (gas chromatography-mass spectrometry) for 6 min for thermal desorption directly to the GC column.

The GC-MS analyses were conducted with an Agilent Technologies (Palo Alto, CA, USA) gas chromatograph model 8890 coupled with a mass selective detector (MSD) model 5977E. HP-5MS capillary column (5% phenyl-methylpolysiloxane, Agilent J and W, Santa Clara, CA, USA) was used for the chromatographic separation. The oven temperature was programmed at 70 °C for 2 min and then increased from 70 to 200 °C (3 °C/min) and held at 200 °C for 15 min at the total run time of 63.333 min. The carrier gas was He (1.0 mL/min). The MSD (mass selective detector) (EI mode) was operated at 70 eV, and the mass range was 30–300 amu. The identification was based on the comparison of the retention indices (RI), determined relative to *n*-alkanes (C_9_–C_25_), with those reported in the literature and our internal database (Department of Organic Chemistry, Faculty of Chemistry and Technology, University of Split) as well as their mass spectra with the authentic compounds available in our laboratory or those spectra listed in mass spectral libraries NIST 17 (D-Gaithersburg, MD, USA) and Wiley W9N08 (Wiley, New York, NY, USA). The percentage composition was computed from the GC peak areas using the normalization method, and the average component percentages were calculated from duplicate GC-MS analyses.

### 2.5. FTIR-ATR Spectroscopy and Spectral Data Processing

The infrared (IR) spectra of collected bee pollen samples were recorded using Cary 660 Fourier transform mid-infrared (FTIR) spectrometer (Agilent Technologies, Palo Alto, CA, USA) coupled with Golden Gate single-reflection diamond Attenuated Total Reflectance (ATR) accessory (Specac). Prior to the spectral analysis, bee pollen samples (pollen loads) were pulverized with porcelain mortar into fine homogenates. To obtain IR spectra of a thin uniform layer of each sample, 3–5 mg of homogenized bee pollen was pressured on a diamond ATR plate using self-leveling sapphire anvil. The absorption spectra were recorded in a mid-infrared region (4000–400 cm^−1^) at nominal resolution of 4 cm^−1^ (at room temperature 24 ± 2 °C). Two replicate spectra (32 scans/spectrum) of each sample were recorded using different aliquots. The spectra were recorded and stored using Resolutions Pro version 5.3.0 software package [36]. Further qualitative interpretation of the bee pollen IR spectra (assignation of molecular vibrations) and corresponding spectral data analysis was performed using Origin version 8.1 [37] based on the spectral atlases and available scientific literature.

### 2.6. Statistical Analyses

Physico-chemical measurement data were analyzed by means of descriptive statistics, as well as classical one-way analysis of variance (ANOVA) and Kruskal–Wallis one-way ANOVA in order to assess the differences between multifloral and unifloral bee pollen samples originating from three climatic–geographical regions (CR, MR, and AR). In addition, physico-chemical parameters of unifloral bee pollen samples of the same botanical origin from different regions were compared using paired sample t test to assess statistical significance of differences among the samples. Above mentioned statistical analyses were performed using statistical software package Statistica-StatSoft v.7 [38] (StatSoft Inc., Tulsa, OK, USA).

## 3. Results

### 3.1. Bee Pollen Classification According to Botanical Origin

In total, 16 multifloral bee pollen samples were collected (6 pooled samples from CR, 6 from MR, and 4 from AR). The pooled bee pollen samples were analyzed as obtained. Afterward, the pollen loads of the same botanical origin were isolated from the pooled samples. Identification of the botanical origin of isolated bee pollen relied upon the confirmation of uniflorality by melissopalynological analysis. In total, 48 unifloral bee pollen samples were identified and subjected to further analyses. Among them, unifloral bee pollen samples originating from 29 different plant species were identified. Distribution of collected unifloral bee pollen samples according to botanical origin and region is presented in Table 1.

### 3.2. Characterization of Bee Pollen by Physico-Chemical Analyses

Physico-chemical analyses were performed on multifloral and unifloral bee pollen samples (containing >30 g necessary for the analyses) isolated from the pooled samples for overall physico-chemical analyses. Bee pollen collected by the honey bees in CR contained six multifloral bee pollen samples (coded as mCR1-mCR6) and nine unifloral bee pollen samples, in MR contained six multifloral bee pollen samples (coded as mMR1-mMR6) and nine unifloral bee pollen samples, while in AR contained four multifloral bee pollen samples (coded as mAR1-mAR4), and three unifloral bee pollen samples. The unifloral bee pollen samples were denoted as corresponding plant species. The results of the physico-chemical analyses are presented in Table 2.

The moisture content in collected multifloral bee pollen samples from CR ranged from 15.01% to 22.40% and the ash content from 1.75% to 2.80%. The protein content ranged from 14.73% to 23.19%, total lipids from 8.74% to even 18.36%, which differed significantly from the other multifloral bee pollen samples from CR. Dominant sugars in analyzed bee pollen were monosaccharides fructose (13.99–20.05%) and glucose (8.92–15.87%), while the most represented disaccharides were sucrose (5.87–22.04%), maltose (1.56–3.85%), melezitose (0.27–1.54%), and raffinose (0.02–0.23%).

The unifloral bee pollen of *Viburnum* spp. contained the lowest moisture content (13.80%) while the highest value was observed in *T. officinale* bee pollen (21.40%). The ash content ranged from 1.15% in *T. officinale* to 3.08% in *C. sanguinea* bee pollen sample. The highest protein content was determined in *A. hippocastanum* bee pollen (27.26%), and the lowest in *T. officinale* (14.02%). Conversely, *T. officinale* bee pollen contained the highest total lipid content (19.04%), while the lowest was determined in *A. hippocastanum* bee pollen (6.20%). Fructose ranged from 10.87% in *A. hippocastanum* bee pollen to 25.65% in *P. rhoeas* bee pollen, the content of glucose in *A. hippocastanum* bee pollen was 8.96%, and 18.80% in *T. officinale* bee pollen, while sucrose ranged from 1.62% in *T. officinale* bee pollen to 20.33% in *P. spinosa* bee pollen. The highest value of other less represented sugars was determined for maltose in *T. officinale* bee pollen (4.33%), and *J. regia* (2.79%) bee pollen.

The moisture content in multifloral bee pollen samples from MR ranged from 13.78% to 22.21%, the ash content from 1.84% to 2.89%, the protein content from 15.59% to 23.29%, and the total lipids from 6.13% to 14.57%. Sugar with the highest content was fructose (19.88%), followed by glucose with 18.70% and sucrose with 15.85%.

Observing the values of unifloral bee pollen samples from MR, the lowest moisture content was found in *P. spinosa* (11.30%), and the highest in *Q. pubescens* samples (26.48%). The ash content ranged from 1.18% in *T. officinale* bee pollen to 3.81% in *P. avium* bee pollen. Proteins were the second largest constituent in the bee pollen after sugars. In *P. tanacetifolia*, bee pollen proteins were found with the content of 26.32%, and the lowest content was recorded in the bee pollen of *T. officinale* (13.90%), which is similar to the value obtained for bee pollen from CR. The highest total lipid content was determined in *T. officinale* bee pollen (17.53%), and the lowest in *F. vulgaris* bee pollen (4.51%). The most represented sugar was fructose in *P. tanacetifolia* bee pollen (24.28%), as well glucose (18.49%) and sucrose in *P. spinosa* bee pollen (18.68%), while other sugars ranged from 0.05% for melezitose in *Q. pubescens* bee pollen to 5.48% for maltose in *M. sativa* bee pollen.

The moisture content in multifloral samples from AR ranged from 14.18% to 22.84% and the ash content from 2.06% to 2.49%. The proteins were represented in the range of 17.21–21.19%, while the total lipids ranged from 8.52% to 10.80%. In this location, the highest value of fructose was recorded (23.88%) compared to the other two investigated locations, while the highest value of glucose and sucrose were 16.80% and 8.50%, respectively.

The unifloral bee pollen of *P. mahaleb* contained the lowest moisture content (12.75%), and the highest one was determined in *C. biennis* sample (17.49%). The highest value of ash (3.09%) and protein content (22.23%) was observed in *P. mahaleb* bee pollen, while in *C. biennis* bee pollen, these values were the lowest (1.56% for ash and 16.60% for protein content). The content of fructose and glucose was the highest in *C. biennis* bee pollen (20.08% and 15.51%, respectively), while the lowest ones were found in *P. mahaleb* bee pollen (14.17% and 11.95%). The highest sucrose content was found in *P. mahaleb* bee pollen (14.37%). Maltose, melezitose and raffinose ranged from 0.09% to 3.38% in various unifloral bee pollen samples.

The results of ANOVA showed that there are no statistically significant differences between multifloral and unifloral bee pollen originating from different regions for most of physico-chemical measurement data (moisture, ash, proteins, total lipids, total sugars, fructose, glucose, sucrose, maltose, raffinose), with an exception of melezitose content that was significantly different (*p* = 0.04). The effects of classical ANOVA and Kruskal–Wallis one-way ANOVA are presented in detail in the Supplementary Materials S1.

Furthermore, the results of one-tail paired sample t test comparing paired sets of physico-chemical measurement data (moisture, ash, proteins, total lipids, total sugars, fructose, glucose, sucrose, maltose, melezitose, raffinose) for bee pollen of the same botanical origin but from different regions (*Q. pubescens*: MR, AR; *T. officinale*: CR, MR; *J. regia*: CR, MR; *P. spinosa*: CR, MR; *Salix* spp.: CR, MR) revealed no statistically significant differences for all physico-chemical parameters tested (*p* < 0.05), as presented in Table S1 in the Supplementary Materials S1.

### 3.3. The Headspace Composition of Unifloral Bee Pollen

The headspace (HS) bee pollen volatile organic compounds (VOCs) were determined by headspace solid-phase microextraction followed by gas chromatography and mass spectrometry analysis (HS-SPME/GC-MS) by using DVB/CAR/PDMS fiber. Since the bee pollen VOCs are located in pollenkitt, the temperature of 60 °C was used for HS-SPME to overcome slow volatilization as the consequence of the presence of lipids in pollenkitt, such as mono-, di- and triglycerides, which can retard the emission of volatiles. Remarkable differences were found among the bee pollen HS VOCs.

The bee pollen collected by the honey bees from CR were the most diverse among all locations and collecting periods, including the bee pollen from *R. acris* L., *F. excelsior* L., *P. sylvestris* L., *A. schoenoprasum* L., *C. biennis* L., *J. regia* L., *Quercus* spp., *A. hippocastanum* L. and *Salix* spp. (Table 3). (*E*,*E*)-Geranyl linalool (49.27%) prevailed in *R. acris* bee pollen HS VOCs along with (*E*)-geranyl acetone (5.46%). Two other abundant compounds were 6-methylhept-5-en-2-one (12.39%) and nonanal (8.92%). Principal components of *F. excelsior* bee pollen headspace were 6-methylhept-5-en-2-one (21.45%), (*E*)-geranyl acetone (16.19%) and nonanal (15.22%). Nonanal (46.70%) predominated in HS VOCs of *P. sylvestris* bee pollen. Several monoterpenes were found as minor constituents such as α-pinene, β-pinene, δ-3-carene, *p*-cymene, γ-terpinene as well as decanal. Benzaldehyde (29.11%), nonanal (14.38%), pentadecane (9.63%) and cyclopent-2-ene-1,4-dione (6.14%) were leading compounds of *A. schoenoprasum* bee pollen headspace. *C. biennis* bee pollen headspace mainly contained hexanoic acid (24.46%) and heneicosane (20.17%), followed by nonanal (8.37%), octanoic acid (8.78%), acetoin (5.06%) and hexanal (4.59%). Nonanal (59.76%) dominated in HS VOCs of *J. regia* bee pollen, followed by hexanal (7.34%), 6-methylhept-5-en-2-one (8.61%) and (*E*)-β-ocymene (5.04%). The major compound of HS VOCs of *Quercus* spp. bee pollen was benzaldehyde (40.34%) and verbenone (13.11%), followed by nonanal (6.98%) and other lower aliphatic compounds. Butanal (20.96%) and benzaldehyde (18.68%) were principal constituents of HS VOCs of *A. hippocastanum* bee pollen. Other abundant compounds were 3-methylbutanal (8.85%), hexanal (5.40%), 2-ethylhexan-1-ol (6.15%) and nonanal (7.84%). Benzaldehyde (27.46%) also prevailed in HS VOCs of *Salix* spp. bee pollen, followed by berbenone (21.44%).

The samples from MR contained the bee pollen of *P. avium* L., *Salix* spp., *T. officinale* L., *P. sylvestris* L., *C. biennis* L., and *Q. pubescens* (Table 4). The bee pollen composition is completely different than in CR due to various plants distribution. Lilac aldehydes were the major compounds of *P. avium* bee pollen headspace: lilac aldehydes (A 14.84%. B 29.30% and D 13.37%) followed by lilac alcohols (A 11.37%. B 13.76% and D 4.21%). HS VOCs of *Salix* spp. bee pollen contained mainly 3-hydroxybutan-2-one (5.47%), pentanal (4.14%), hexanal (7.02%), heptanal (5.17%) and nonanal (9.28%). Another abound group of compounds belongs to lilac aldehydes (A 6.06%; B 10.43% and D 3.94%). Pentadecane was also abundant with 10.29%. Lower aliphatic compounds were abundant in *T. officinale* bee pollen headspace being dominated by 3-methylpentanal (10.37%), 2-methylbutanal (5.42%) and 3-methylbutanal (4.57%). Another major compound was benzaldehyde (28.98%). Lilac alcohols were present at minor percentages (A 3.85%. B 6.42% and D 2.48%). The predominant compound in HS VOCs of *P. sylvestris* bee pollen was pentadecane (51.16%). Other abundant compounds were acetic acid 10.10%, ethanol 6.93%, β-phellandrene (5.69%) and nonanal (8.09%). HS VOCs of *C. biennis* bee pollen mainly constituted heneicosane (18.23%), acetic acid (21.34%), acetoin (11.87%) and ethanol (7.17%). Lower percentages of benzaldehyde and phenylacetaldehyde were found. The principal compounds of *Q. pubescens* bee pollen headspace were: acetic acid (21.69%), ethanol (9.44%), acetoin (7.07%), pentanal (11.85%), heptanal (5.37%), nonanal (15.57%) and (*E*,*E*)-octa-3,5-dien-2-one (5.08%).

The bee pollen collected from AR was composed of the bee pollen from *Q. pubescens*, *Malus* spp., *S. vulgaris*, *R. lutea*, *C. biennis*, and *P. mahaleb* (Table 5). The bee pollen HS of *Q. pubescens* contained, as the major compounds, acetic acid (6.93%), acetoin (5.05%), hexanal (4.88%), heptanal (4.46%), benzaldehyde (4.88%), 6-methylhept-5-en-2-one (5.81%), phenylacetaldehyde (5.46%) and nonanal (28.67%). *Malus* spp. bee pollen HS contained, as dominant compounds, pentanal (1.46%), hexanal (3.56%), heptanal (2.19%) and nonanal (15.53%) followed by 6-methylhept-5-en-2-one (8.33%). Other abundant compounds were benzene derivatives benzaldehyde (6.36%), phenylacetaldehyde (5.69%) and 2-phenylethanol (2.60%). Acetic acid (9.30%) and acetoin (3.99%) were also detected. HS VOCs of *S. vulgaris* bee pollen were composed of the following major compounds: heptan-2-ol (30.63%), acetic acid (9.27%), nonanal (4.60%) and lilac aldehyde B (5.20%). The major compounds of *R. lutea* bee pollen HS were: acetic acid (19.09%), 6-methylhep-5-en-2-one (9.56%), benzaldehyde (5.60%), phenylacetaldehyde (11.79%) and nonanal (8.14%). *C. biennis* bee pollen HS VOCs contained acetoin as the major compound (19.41%). Another group of abundant compounds were lilac aldehydes (A 5.61%, B 9.00% and D 3.28%). Lower aliphatic aldehydes were also present (pentanal 5.83%, hexanal 5.22% and nonanal 3.65%) as well as 2-ethylhexan-1-ol (5.41%). The bee pollen of *P. mahaleb* among major HS constituents contained lilac aldehydes (A 14.69%, B 25.04% and D 14.51%), accompanied by lilac alcohol A (2.99%). Another abundant compound was nonanal (5.19%).

### 3.4. FTIR-ATR Spectral Profiling

**Figure 1:**
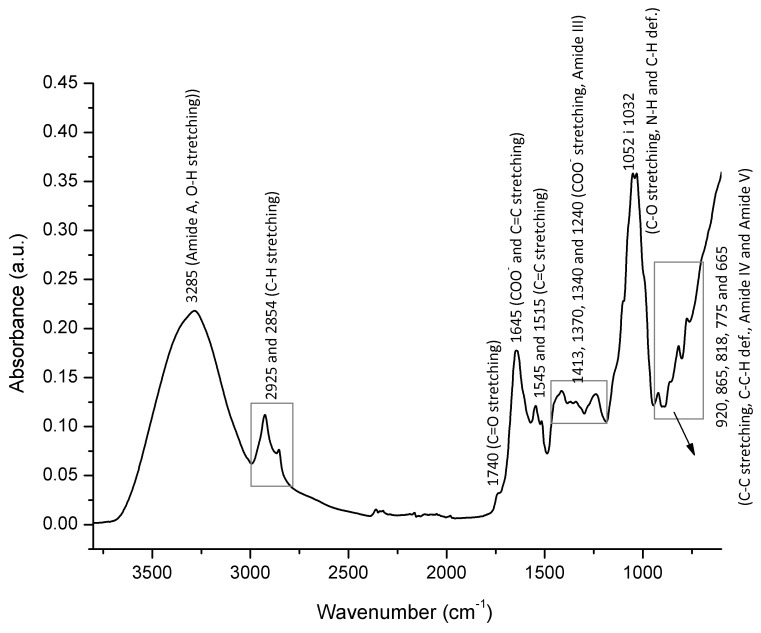
Characteristic FTIR-ATR spectrum of bee pollen with the assignation of major underlying molecular vibrations.

A characteristic FTIR-ATR spectrum of bee pollen (obtained as an average spectrum of the entire set of multifloral and unifloral bee pollen spectra) with assignation of major underlying molecular vibrations is presented in Figure 1.

A typical FTIR-ATR spectrum of bee pollen is characterized by the broad absorption observed in the spectral range from 3600 to 3000 cm^−1^ with an absorption maximum at 3285 cm^−1^, which can be assigned to both N–H stretching vibrations of the protein structure (amide A band) [39,40], and O–H stretching vibrations of carbohydrates (primarily glucose, fructose, and sucrose) and water. Two IR absorptions, the medium intensity absorption band observed at 2925 cm^−1^ and the weaker one at 2854 cm^−1^, correspond to C–H stretching (aliphatic C–H groups) and can be described as non-specific IR signals given that they may be related to the molecular vibrations of numerous bee pollen constituents, such as lipids, fatty acids, carbohydrates, cellulose, and other long-chain structures [11,24,25,26,27].

The spectral region between 1800 and 700 cm^−1^ (fingerprint region) is populated by a series of absorption bands that reflect unique spectral pattern of bee pollen. A weak absorption band peaking at 1740 cm^−1^ occurs primarily due to the carbonyl group C=O stretching vibrations of the of the ester bond [39] in lipid-based bee pollen constituents. A medium intense absorption band observed at 1645 cm^−1^ is characterized by overlapping effects of several vibrations related to lipids, proteins and water. The lipids are represented by absorption due to COO^–^ and C=C stretching given that COO^–^ asymmetric stretching (RCOO^–^–group) typically absorb between 1610 and1550 cm^−1^, and C=C stretching between 1680 and1600 cm^−1^ [39]. This band position can also be assigned to amide I, an intense absorption band in proteins (β-sheet) primarily governed by the stretching vibrations of the C=O (70–85%) and C–N groups (10–20%) that can be attributed to the protein fraction of bee pollen. Absorption at 1645 cm^−1^ also belongs to well-known molecular vibrations of water, H–O–H deformation vibration, namely.

The band at 1645 cm^−1^ is followed by the lower intensity analyte signals occurring at 1545 and 1515 cm^−1^ that are attributed to C=C stretchings of aromatic structures, primarily lipid-based. It can be assumed that protein-related amide II band that comprises N–H bending and C–N stretching vibrations and typically absorb between 1510 and 1580 cm^−1^, is overlapped by the former C=C stretchings.

The spectral region between 1490 and 1190 cm^−1^ is populated by a series of low intensity analyte signals primarily attributed to proteins and sugars. The absorption bands peaking at 1413, 1370 and 1340 cm^−1^ can be linked to the protein side chain COO^–^ stretching vibrations, while the absorption at 1240 cm^−1^ occurs due to the amide III band, which comprises 30% of C–N stretching, 30% of N–H bending, 10% of C–O stretching, and 10% of O=C–N bending vibrations (the rest belongs to other vibrations) [39]. In this spectral region, mentioned protein-related absorptions overlap less intensive sugar vibrations: weak bands peaking at 1413 and 1340 cm^−1^ that are assigned to C–O–H deformation of glucose and fructose, respectively, while the band at 1240 cm^−1^ occurs due to CH_2_ bending of glucose [41,42,43].

**Figure 2:**
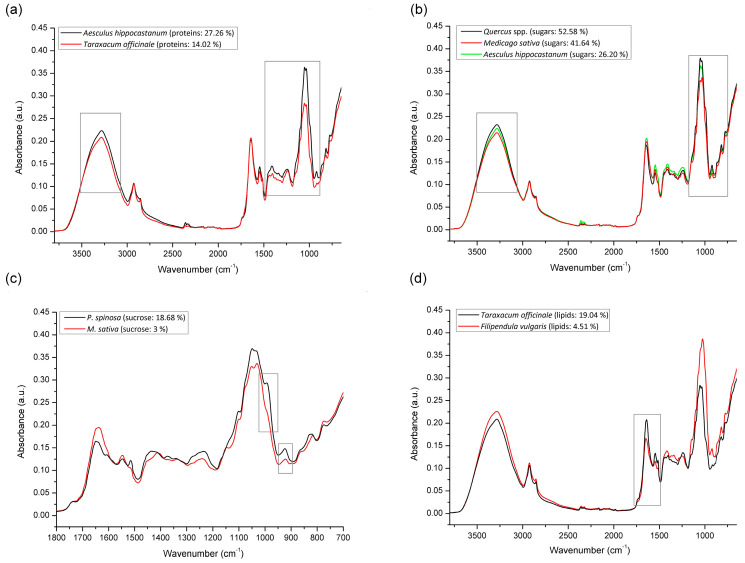
Comparative spectral features of bee pollen (FTIR-ATR spectra of unifloral bee pollen containing the highest vs. the lowest content of particular organic compound) with accentuated spectral regions indicative for the content of: proteins (**a**), sugars (**b**), sucrose (**c**), and lipids (**d**).

The most prominent absorption envelope observed in the FTIR-ATR spectrum of bee pollen occurs between 1170 and 950 cm^−1^, and it is followed by less intensive vibrations occurring between 950 and 750 cm^−1^. These spectral regions are also characterized by the overlapping spectral effects covering both sugar and protein related functional group vibrational modes. A weak absorption band observed at 1100 cm^−1^ (along with shoulder at 1077 cm^−1^) can be assigned to the stretching vibration of endo C–O bonds of the most predominant sugars in bee pollen, glucose and fructose [41,43], as well as C–H and N–H in-plane deformation vibrations of aromatic structures related to proteins. The most prominent absorption bands observed in the bee pollen spectrum arising at 1052 and 1032 cm^−1^ are attributed to numerous overlapping signals of sugars and proteins, stretching C–O vibrations, peaking at 1052 cm^−1^ for fructose, and at 1032 cm^−1^ for glucose [43], as well as N–H and C–H deformation vibrations in relation to proteins. Less intensive absorption occurring at 993 cm^−1^ is known as the characteristic sucrose bend related to glycosidic linkage [39]. This was additionally confirmed by a comparative analysis of unifloral bee pollen containing high vs. low amount of sucrose presented in the latter Figure 2c.

There are five weak absorptions occurring in the spectral range from 950 to 750 cm^−1^. The bands occurring at 920 and 865 cm^−1^ are assigned to skeletal C–C stretching vibrations of monosaccharide’s carbon backbone, for glucose and fructose, respectively, while the bands peaking at 818 and 776 cm^−1^ correspond to the deformation C–C–H vibrations of fructose [40]. Sucrose C–C stretchings also contribute to the signal observed at 920 cm^−1^. Vibrations related to the protein structure are also observable in this spectral region and overlap with some of the above mentioned sugar-specific signals; the band at 665 cm^−1^ corresponds to O=C–N bending (amide IV), and the signal at 776 cm^−1^ to out-of-plane N–H bending (amide V) [39,40].

Comparative spectral features with regard to spectral regions indicative for the detection of higher amounts of particular organic compounds (proteins, sugars, lipids) are presented in Figure 2. As presented in Figure 2a showing a comparative overview of IR spectra of *A. hippocastanum* bee pollen containing the highest vs. *T. officinale* bee pollen with the lowest protein content, it is obvious that spectral features indicative for the protein fraction are represented with a series of absorption bands occurring in the spectral region between 1480 and 950 cm^−1^ (integral absorption intensities are higher in the spectrum of *A. hippocastanum* bee pollen). However, within this wide region, spectral range 1480–1185 cm^−1^ can be identified as highly specific for proteins (due to series of amide III bands) while the spectral range from 1185 to 950 cm^−1^ may also reflect signals due to strong sugar absorptions (primarily glucose, fructose and sucrose; Figure 2b,c) appearing along with protein-related absorptions.

The overlapping effects of sugar and protein signals in 1185–950 cm^−1^ spectral region does not allow clear distinguishing of predominant compounds when both are represented in high amounts (the same goes for the spectral range from 3600 to 3000 cm^−1^). Figure 2b represents this combination: the spectrum of *Quercus* spp. bee pollen containing a high sugar content (52.58%) shows spectral differences that are not particularly pronounced compared to *A. hippocastanum* bee pollen, which contains only 26.2% of sugar, but also a significant amount of proteins (27.26%) whose bands overlap in these region. Still, mentioned spectral differences becomes more prominent (in terms of lower absorption intensities) as the protein content decreases, such as in the case of *M. sativa* bee pollen that contains less of both sugars and proteins (41.61% and 23.56%, respectively) compared to *Quercus* spp. bee pollen. Furthermore, Figure 2c shows *P. spinosa* bee pollen with the highest amount of sucrose (18.68%) vs. *M. sativa* bee pollen with underrepresented sucrose content (3%). The results presented in this figure have revealed that the intensities of bands occurring at 993 and 920 cm^−1^, are notably higher in the spectrum of *P. spinosa* bee pollen, which indicate that these bands are sucrose-specific (the proportion of other constituents is similar in these two bee pollen samples). Moreover, the vibration at 993 cm^−1^ transforms into a shoulder form in case when sucrose in underrepresented.

As shown in Figure 2d representing FTIR spectra of *T. officinale* bee pollen containing the highest vs. *F. vulgaris* bee pollen with the lowest lipid content, spectral features indicative for lipids are observable in the spectral region between 1760 and 1500 cm^−1^ comprising four lipid-related absorptions at 1740, 1645, 1545 and 1515 cm^−1^. The results of the spectral analyses have revealed that this region is the only lipid-specific region; other spectral dissimilarities observed in Figure 2d (and presented in previous Figure 2a–c) reflect the compositional variations related to sugars and proteins. The same observations were confirmed in case of multifloral bee pollen samples.

**Figure 3:**
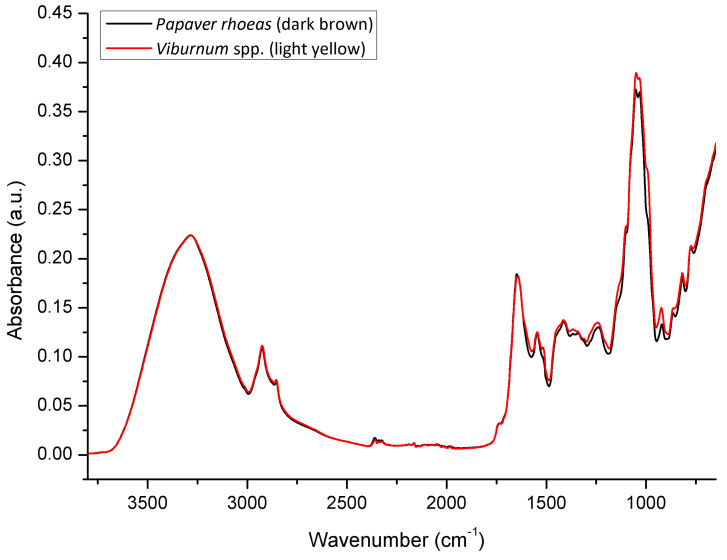
Comparative spectral features of bee pollen with regard to pigmentation (FTIR-ATR spectra of dark colored *P. rhoeas* vs. light colored *Viburnum* spp. bee pollen).

The spectral analysis also revealed that there are no molecular vibrations indicative for pigmentation of bee pollen, as the dark colored (dark brown) *P. rhoeas* bee pollen exhibits almost equal spectral pattern as the light colored (light yellow) *Viburnum* spp. bee pollen (Figure 3). Spectral similarity is a consequence of similar overall chemical composition of these two bee pollen samples.

Descriptive sheets for unifloral bee pollen collected from three Croatian regions (continental-CR, mountain-MR, and Adriatic-AR) representing visual identity (color), nutritional value (content of ash, proteins, total lipids, total sugars, fructose, glucose and sucrose; expressed as a percentage of the total weight), and FTIR-ATR spectra (fingerprint region: 1800–650 cm^−1^) are provided in Supplementary Materials S2.

## 4. Discussion

Knowledge of the chemical composition plays an important role in determining the nutritional value of bee pollen, especially for human use. The physico-chemical properties of bee pollen can be affected by processing techniques [44,45], as well as storage conditions [46], but the overall quality of bee pollen primarily depends on biological factors. Freshly collected bee pollen may contain 10% to 30% moisture [4,12,47,48,49,50], which is in agreement with the data obtained in this study, where the moisture content ranged from 11.30% to 26.48%. The fresh bee pollen has more important nutritional value for human use compared to dried bee pollen, which contains less than 8% of moisture [4]. However, the high content of moisture in fresh bee pollen makes it an ideal culture for different microorganisms, particularly yeasts and molds, and to preserve the good quality of bee pollen, it should be harvested daily from pollen traps and stored in deep freezer under −18 °C until consumption.

Ash (minerals) is important for nutritional value of bee pollen, but its content can be largely affected by climate, geographical and botanical origin. However, soil type has the main influence on the mineral content of bee pollen [51,52]. In average, bee pollen contains 0.5–6.5% of ash [4,26,48,50,53,54,55,56,57]. The results obtained in the present study are in compliance with above mentioned studies, as the ash content ranged from 1.15% to 3.81%. The mineral composition is stable, and it does not change significantly in the drying process of bee pollen, as reported by De Melo et al. [44] and Isik et al. [45].

Numerous studies have covered investigation of various physico-chemical parameters of multifloral bee pollen, including protein content [4,12,49,52]. Although multifloral bee pollen contains pollen grains of different plant species, its total protein content is on average not always significantly higher compared to unifloral bee pollen. The results presented in this study confirmed this; the unifloral bee pollen from *Prunus* species, *P. tanacetifolia* and *A. hippocastanum* contained a higher protein content compared to other investigated unifloral and multifloral bee pollen samples. The protein content showed significant variations among the pollen samples ranging from 14.23% to 23.29% in the multifloral bee pollen, and from 14.02% in *T. officinale* bee pollen to 27.26% in *A. hippocastanum* bee pollen. The obtained data are in agreement with the results reported by Isopescu et al. [26], Kostić et al. [56], Barajas et al. [57], Taha et al. [58], and Aličić et al. [59], higher than those obtained by Orzalez Villanueva [60], and lower compared to reports provided by Asmae et al. [50], Estevinho et al. [61], and Liolios et al. [62].

Glucose, fructose and sucrose were the major components in investigated bee pollen samples, and they showed great variations, especially among unifloral bee pollen samples. As presented in Table 2, fructose ranged from 10.79% to 23.88%, glucose from 8.92% to 18.70% and sucrose from only 2.62% to 22.04% in multifloral bee pollen samples. These values are in compliance with findings reported Qian et al. [63], Bobis et al. [64], Martins et al. [65], and Bertoncelj et al. [66]. In unifloral samples fructose ranged from 10.87% in *T. officinale* bee pollen to 25.65% in *A. hippocastanum* bee pollen, glucose from 8.96% also in *A. hippocastnum* bee pollen to 19.86% in *Q. pubescens* bee pollen, and sucrose from 1.62% in *T. officinale* bee pollen to 20.33% in *P. spinosa* bee pollen. Comparing obtained values to the ones of some unifloral bee pollen samples from available literature, such as *T. officinale* and *Prunus* spp. from Romania [12], *Salix* spp. from Serbia [56], *P. tanacetifolia* and *Salix* spp. from Slovenia [66,67], and *T. officinale* and *Salix* spp. from Bosnia and Herzegovina [59], certain differences in observed nutritional values can be noticed. Since these countries have similar climatic conditions, this is additional evidence that the nutritional value of bee pollen depends not only on the botanical origin and climate conditions, but also on other factors, such as soil type and bee pollen collecting period and processing.

Bee pollen has a specific taste and aroma, and it can therefore be unpleasant for consumers (depending on consumers’ preferences). In this case, it can be mixed with other food products, such as honey, juices and jams for better acceptability [68]. According to Campos et al. [69], recommended daily intake is 20–30 g, but this strongly depends on the botanical origin of the bee pollen. Therefore, knowledge of the nutritional value of bee pollen is of great importance for use in the human diet, especially as a food supplement [5].

To identify VOCs derived specifically from pollen, hand-collected pollen is preferred over the more readily obtained bee-collected pollen [70], which may contain various pollen contaminants and show alterations in VOCs due to added bee secretions and nectar. It was found that the principal constituents in the headspace of honey bee workers are aldehydes from the chain length C_9_ to C_17_ [71], and a variety of lower aliphatic aldehydes were found in the bee pollen headspace (Table 3, Table 4 and Table 5) dominated by nonanal. The large amounts of these compounds found in the headspace of honey bee workers suggest that they do not originate from the cuticle (lower aldehydes were found in traces). A possible source may be alkenes and alkadienes degradation caused by oxygen, heat or sunlight [72]. The oxidation of unsaturated hydrocarbons is also known from the cuticle of other Hymenoptera [73]. From the bees’ headspace, these compounds can be incorporated in the lipids in pollenkitt. However, their other origin cannot be excluded, but the fact that lower aldehydes (especially C_9_) were present in almost all bees collected bee pollen support possible contribution from the bees. The majority of compounds on the cuticle of honey bees are long-chain alkanes, branched alkanes, alkenes and esters [74]. In the bee pollen headspace, only pentadecane and heneicosane appeared in several samples as predominant compounds, indicating their plant origin than transfer from the bees, since in that case, higher abundance will be noticed in all the samples. However, hydrocarbons such as hexadecane, octadecane and heneicosene, are likely to be the key components for nestmate recognition [75].

3-Methylbutanal, and 2-methylbutanal, known Strecker aldehydes derived from valine, leucine, and isoleucine [70], were found occasionally (particularly in *Salix* spp., *Quercus* spp., *A. hippocastanum* and *T. officinale* bee pollen headspace). Dimethyl sulfide was rarely present (only in *Malus* spp. and *R. lutea* bee pollen headspace) and probably produced by S-methylmethionine degradation [70].

Several benzene derivatives were found, the major ones were benzaldehyde (in all samples, particularly in *Quercus* spp., *A. schoenoprasum, T. officinale* and *Salix* spp. bee pollen headspace) and phenylacetaldehyde (particularly in *R. lutea* bee pollen headspace). These compounds have frequently been found in different honey types [76]. Phenylacetaldehyde can be generated from phenylalanine by the enzyme catalysis or Strecker degradation [76]. Benzaldehyde was found in *Salix* spp. honey [76,77].

Terpenes found in bee pollen are probably related to plant and pollen origin. Similar as in different honey types [78], the great majority of found terpenes in the samples collected in location Otočac and Senj, especially the bee pollen from *P. mahaleb* and *P. avium* (Table 3 and Table 4), were monoterpenes, particularly linalool derivatives such as isomers of lilac alcohol or lilac aldehyde. Starting from linalool, a variety of compounds can be formed. Direct hydroxylation of linalool forms (*E*)-8-hydroxylinalool that can be transformed via (*E*)-8-oxolinalool by the enzymatic ω-oxidation to lilac alcohols that, by further oxidation, yield lilac aldehydes [78]. Alternatively, epoxidation of linalool produces 6,7-epoxylinalool, which undergoes further reactions to form isomeric furanoid linalool oxides and anhydrolinalool oxides, can further yield lilac alcohols. Terpenes were the most abundant headspace volatiles in *P. mahaleb* honey [79] such as linalool (4.0–6.6%), three isomers of lilac aldehyde (1.0–11.7%), three isomers of lilac alcohol (0.1–1.2%), and *cis*- and *trans*-linalool oxide (0.6–3.1%). However, from these compounds, only traces of *cis*- and *trans*-linalool oxide were found among essential oil from flowers, leaves, bark and wood of *P. mahaleb* [80]. Conversely, linalool derivatives were not found in *P. avium* [81]. Lower percentages of lilac aldehydes and alcohols were found in *Salix* spp. honey [82]. Verbenone, bicyclic monoterpenes with pinane skeletons were identified with significant percentages in *Salix* spp. and *Quercus* spp. bee pollen from CR. It was found previously in *Salix* spp. honey headspace at a lower abundance [78]. 6-Methylhept-5-en-2-one is a frequently reported natural product [83] and can be viewed as degraded terpenoid compound. It was found almost in all samples, particularly in *R. acris* and *F. excelsior* bee pollen from CR. Several monoterpenes were found as minor constituents in *P. sylvestris* bee pollen headspace, such as α-pinene, β-pinene, δ-3-carene, *p*-cymene, or γ-terpinene. Those compounds are well-known, major constituents of the essential oil of *P. sylvestris* [84]. (*E*)-geranyl acetone in *F. excelsior* and *R. acris* bee pollen headspace and diterpene (*E*,*E*)-geranyl linalool was predominant in *R. acris*.

Bee pollen represents a complex organic mixture of various polymeric molecules with predominant constituents covering all major classes of biological macromolecules-proteins, lipids and carbohydrates. Such compositional complexity reflects in a complex FTIR-ATR spectrum of bee pollen exhibiting a broad variety of molecular vibrations characterized by numerous overlapping spectral features.

The first valuable insight into the bee pollen IR spectrum was reported by Anjos et al. [11], who reported on the usefulness of this technique for studying overall chemical composition of bee pollen. Further investigations covered analysis of unifloral rape bee pollen [24], bee pollen of 13 botanical families and 17 genus [25], and investigation of unifloral Romanian bee pollen [26]. The most comprehensive one was provided by Castiglioni et al. [25], who conducted insightful research on morphological, physico-chemical and spectroscopic properties of unifloral bee pollen. Mentioned authors have provided various assignations for the same absorption bands occurring in the bee pollen IR spectrum, many of which were unspecific or incomplete, i.e., described in a pooled form without clearly assigning the particular band to a corresponding molecular vibration. An example for this is a description of three absorption bands occurring in the spectral region between 1700 and 1500 cm^−1^ were previously described as cellulose and protein-relating regions [24,25,26]. Significant contribution of cellulose and hemicellulose in the overall spectral features cannot be expected considering the insignificant amount of these constituents in the total composition of bee pollen. Several incorrect assignations have also been provided, such as ketone- and quinine-related assignations [26].

In this study, the formerly described assignations were complemented, and overlapping spectral effects have been additionally explained and elucidated based on the comparative overview of spectral data in relation to physico-chemical measurements (protein, lipid and sugar content).

Based on the overall spectral analysis demonstrated in this study, it is not possible to predict or quantify individual pollen constituents based on spectral data due to numerous overlapping spectral effects related to the functional group vibrations of proteins, lipids and sugars that were observed in the FTIR spectra of various unifloral and multifloral bee pollen samples. However, integral spectral profiles of each bee pollen IR spectrum provide information on its overall composition and prevalence of individual constituents. This enables rapid preliminary screening of the relative amounts of certain compounds in bee pollen.

A total of 16 unifloral bee pollen types have been characterized in this study by means of physico-chemical, chromatographic and spectroscopic analytical tools. Compared to previous studies, 10 new unifloral bee pollen types were described (*P. spinosa*, *Viburnum* spp., *A. hippocastanum*, *Q. pubescens*, *J. regia*, *F. vulgaris*, *M. sativa*, *P. tanacetifolia*, *C. biennis*, and *P. mahaleb*) given that previous reports covered research (using various analytical methods) on *Quercus* spp. [11,53], *Salix* spp. [56], *T. officinale* [12,53], *Prunus* spp. [11,12], *C. sanguinea* [25], and *P. rhoeas* bee pollen [25,52,53].

## 5. Conclusions

The results of this study confirmed great variability of the chemical composition of honey bee pollen, which strongly depends on the botanical origin of plant species from which it was collected. Distinctive differences were found among the variety of bee pollen samples collected from three different climatic-geographical regions in Croatia (continental, mountain, and Adriatic), as well among the same region, although statistically significant differences were confirmed only for melezitose and sucrose content. The highest diversity of bee pollen according to botanical origin was observed in the continental region (9 bee pollen types) and mountain region (9 bee pollen types), while bee pollen from Adriatic region showed less diversity (3 bee pollen types). The most distinctive unifloral bee pollen with regard to its physico-chemical properties was *A. hippocastanum* (the highest protein content: 27.26%), *Quercus* spp. (the highest total sugar content: 52.58%), *T. officinale* (the highest total lipid content: 19.04%), and *P. avium* (the highest ash content: 3.81%).

Remarkable differences were found among the bee pollen HS VOCs. The major ones were lower aliphatic compounds, monoterpenes and benzene derivatives. Aldehydes C_9_ to C_17_ are principal constituents in the headspace of honey bee workers with possible contribution to the samples, since aldehydes (particularly nonanal) were present in almost all samples. Several benzene derivatives were found, the major ones were benzaldehyde in *T. officinale* and *Salix* spp. bee pollen headspace and phenylacetaldehyde in *R. lutea* bee pollen headspace. Terpenes found (especially from the bee pollen from *P. mahaleb* and *P. avium*) were mainly linalool derivatives (such as isomers of lilac alcohol or lilac aldehyde), which are probably related to plant and pollen origin. Linalool and benzene derivatives are well-known constituents of different honey types.

FTIR-ATR analysis revealed unique spectral profiles of analyzed bee pollen, exhibiting its overall chemical composition arising from molecular vibrations related to major biological macromolecules in bee pollen-proteins, lipids and carbohydrates (sugars). The analysis of these integral spectral patterns of different bee pollen types enables preliminary screening of the relative amounts of certain compounds in bee pollen, i.e., prevalence of individual constituents.

Compared to previous research reports, a total of 16 new unifloral bee pollen types have been characterized by means of both physico-chemical and instrumental analytical tools (chromatographic and spectroscopic). The findings presented in this study complement the current knowledge on the chemical composition and nutritional value of bee pollen, but also provide new insights in terms of headspace composition and FTIR profiles of unifloral bee pollen. Moreover, the dataset on HS VOCs represents the first record on the volatile compounds determined in unifloral bee pollen. A comprehensive set of chemical data on bee pollen presented in this study may contribute to the assessment of its nutritional value in general but may also serve as a basis or a supplement for the establishment of quality criteria for bee pollen on the national and international level. Future trends and developments in bee pollen analysis should be focused on standardization, starting with building the proper physico-chemical reference profile of bee pollen. Along with these trends covering bee pollen quality criteria, future investigations should also be focused on the development of fast, reliable, easy-to-use, and low-cost analytical methods for routine analysis of bee pollen.

## Figures and Tables

**Table 1:** Distribution of collected unifloral bee pollen samples according to botanical origin and region (listed according to the blooming appearance of corresponding plant species).

Region	Botanical Origin
Continental region–CR	willow (*Salix* spp.), oak (*Quercus* spp.), horse chestnut (*Aesculus hippocastanum* L.), blackthorn (*Prunus spinosa* L.), common dandelion (*Taraxacum officinale* L.), viburnum (*Viburnum* spp.), Persian/English walnut (*Juglans regia* L.), common ash (*Fraxinus excelsior* L.), rough hawk’s beard (*Crepis biennis* L.), meadow buttercup (*Ranunculus acris* L.), chives (*Allium schoenoprasum* L.), Scots pine (*Pinus sylvestris* L.), common poppy (*Papaver rhoeas* L.), common dogwood (*Cornus sanguinea* L.), black locust (*Robinia pseudoacacia* L.), blackberry (*Rubus* spp.)
Mountain region–MR	blackthorn (*Prunus spinosa* L.), common dandelion (*Taraxacum officinale* L.), wild cherry (*Prunus avium* L.), willow (*Salix* spp.), rough hawk’s beard (*Crepis biennis* L.), downy oak (*Quercus pubescens* Willd.), Persian/English walnut (*Juglans regia* L.), Scots pine (*Pinus sylvestris* L.), dropwort (*Filipendula vulgaris* Moench), alfalfa (*Medicago sativa* L.), fiddleneck (*Phacelia tanacetifolia* Benth.)
Adriatic region–AR	rough hawk’s beard (*Crepis biennis* L.), downy oak (*Quercus pubescens* Willd.), mahaleb cherry/St. Lucie’s cherry (*Prunus mahaleb* L.), common poppy (*Papaver rhoeas* L.), perfoliate alexanders (*Smyrnium perfoliatum* L.), yellow sweet clover (*Melilotus officinalis* (L.) Pall.), spiny plumeless thistle (*Carduus acanthoides* L.), mouse-ear chickweed (*Cerastium* spp.), wild/yellow mignonette (*Reseda lutea* L.), bladder campion (*Silene vulgaris* (Moench) Garcke), apple (*Malus* spp.)

**Table 2:** Physico-chemical measurements (%) of multifloral and unifloral bee pollen samples collected from continental, mountain and Adriatic region (*n* = 2) *.

	Sample	Moisture	Ash	Proteins	Total Lipids	Total Sugars	Fructose	Glucose	Sucrose	Maltose	Melezitose	Raffinose
**Continental region** **Multifloral**	mCR1	15.940 ± 0.028	2.796 ± 0.034	23.19 ± 0.16	8.536 ± 0.059	43.25 ± 0.69	10.79 ± 0.12	8.915 ± 0.081	22.04 ± 0.00	0.650 ± 0.025	0.853 ± 0.057	-
	mCR2	16.045 ± 0.021	2.561 ± 0.051	19.20 ± 0.57	9.47 ± 0.10	38.76 ± 1.14	13.99 ± 0.20	11.913 ± 0.010	10.99 ± 0.96	1.555 ± 0.030	0.302 ± 0.039	-
	mCR3	15.010 ± 0.127	1.75 ± 0.00	14.73 ± 0.17	18.36 ± 0.28	49.361 ± 0.020	19.69 ± 0.36	15.87 ± 0.21	8.91 ± 0.41	3.116 ± 0.042	1.54 ± 0.11	0.23 ± 0.11
	mCR4	22.395 ± 0.049	2.577 ± 0.055	16.75 ± 0.23	9.928 ± 0.010	45.01 ± 0.29	20.05 ± 0.69	14.94 ± 0.16	5.87 ± 0.66	3.85 ± 0.15	0.298 ± 0.045	-
	mCR5	18.325 ± 0.049	2.736 ± 0.061	17.24 ± 0.12	8.74 ± 0.22	45.872 ± 0.061	20.03 ± 0.38	14.05 ± 0.31	8.30 ± 0.12	3.21 ± 0.53	0.267 ± 0.016	0.02 ± 0.00
	mCR6	16.050 ± 0.057	2.752 ± 0.034	18.91 ± 0.14	8.827 ± 0.051	44.14 ± 1.11	19.09 ± 0.55	14.61 ± 0.67	8.52 ± 0.23	1.878 ± 0.074	-	0.055 ± 0.041
	**Mean**	**17.29 ± 0.06**	**2.53 ± 0.04**	**18.34 ± 0.23**	**10.64 ± 0.12**	**44.40 ± 5.55**	**17.27 ± 0.38**	**13.38 ± 0.24**	**10.77 ± 0.40**	**2.38 ± 0.14**	**0.65 ± 0.05**	**0.10 ± 0.05**
	**Minimum**	**15.01 ±** **0.13**	**1.75 ± 0.00**	**14.73 ±0. 17**	**8.54 ± 0.06**	**38.75 ± 1.14**	**10.79 ± 0.12**	**8.92 ± 0.08**	**5.87 ± 0.66**	**0.65 ± 0.03**	**0.27 ± 0.02**	**0.02 ± 0.00**
	**Maximum**	**22.40 ± 0.05**	**2.80 ± 0.03**	**23.19 ± 0.16**	**18.36 ± 0.28**	**49.36 ± 0.02**	**20.05 ± 0.69**	**15.87 ± 0.21**	**22.04 ± 0.00**	**3.85 ± 0.15**	**1.54 ± 0.11**	**0.23 ± 0.08**
**Continental region** **Unifloral**	*T. officinale*	21.395 ± 0.035	1.151 ± 0.010	14.019 ± 0.011	19.038 ± 0.027	43.61 ± 0.20	18.53 ± 0.31	18.803 ± 0.025	1.615 ± 0.023	4.33 ± 0.10	0.247 ± 0.027	0.085 ± 0.013
	*Salix* spp.	15.27 ± 0.11	2.971 ± 0.021	20.409 ± 0.083	7.475 ± 0.029	49.49 ± 0.53	16.71 ± 0.37	13.95 ± 0.36	15.09 ± 0.13	1.58 ± 0.17	2.154 ± 0.053	0.01 ± 0.00
	*P. spinosa*	14.005 ± 0.035	2.925 ± 0.025	23.93 ± 0.80	8.57 ± 0.12	41.77 ± 1.84	10.97 ± 0.12	9.06 ± 0.68	20.33 ± 0.51	0.54 ± 0.11	0.874 ± 0.056	-
	Viburnum spp.	13.795 ± 0.011	2.836 ± 0.025	23.52 ± 0.65	8.32 ± 0.11	45.52 ± 0.24	14.31 ± 0.38	11.23 ± 0.45	17.32 ± 0.82	1.12 ± 0.34	1.540 ± 0.079	-
	*A. hippocastanum*	14.175 ± 0.078	2.94 ± 0.11	27.26 ± 0.31	6.199 ± 0.082	26.20 ± 1.05	10.87 ± 0.45	8.96 ± 0.59	5.196 ± 0.016	0.95 ± 0.00	0.224 ± 0.078	-
	*Quercus* spp.	16.39 ± 0.00	2.918 ± 0.034	21.08 ± 0.33	7.98 ± 0.12	52.58 ± 0.60	17.75 ± 0.23	14.644 ± 0.013	15.79 ± 0.43	1.648 ± 0.041	2.154 ± 0.046	0.596 ± 0.079
	*J. regia*	13.900 ± 0.042	1.829 ± 0.025	14.367 ± 0.082	17.04 ± 0.26	50.20 ± 0.19	19.875 ± 0.060	16.001 ± 0.058	9.582 ± 0.088	2.79 ± 0.13	1.688 ± 0.018	0.265 ± 0.075
	*P. rhoeas*	18.150 ± 0.028	2.071 ± 0.026	22.72 ± 0.26	7.495 ± 0.043	49.96 ± 0.42	25.653 ± 0.091	18.101 ± 0.064	4.08 ± 0.33	2.13 ± 0.12	-	-
	*C. sanguinea*	16.580 ± 0.057	3.081 ± 0.034	17.74 ± 0.19	10.231 ± 0.042	40.14 ± 0.78	16.37 ± 0.58	12.22 ± 0.67	8.47 ± 0.59	2.70 ± 0.13	0.353 ± 0.037	0.03 ± 0.02
	**Mean**	**15.96 ± 0.04**	**2.52 ± 0.03**	**20.56 ± 0.30**	**10.26 ± 0.09**	**44.39 ± 0.65**	**16.78 ± 0.29**	**13.66 ± 0.32**	**10.83 ± 0.33**	**1.98 ± 0.13**	**1.15 ± 0.05**	**0.20 ± 0.04**
	**Minimum**	**13.80 ± 0.01**	**1.15 ± 0.01**	**14.02 ± 0.01**	**6.20 ± 0.08**	**26.20 ± 1.05**	**10.87 ± 0.45**	**8.96 ± 0.59**	**1.62 ± 0.02**	**0.54 ± 0.11**	**0.22 ± 0.08**	**0.01 ± 0.00**
	**Maximum**	**21.40 ± 0.04**	**3.08 ± 0.03**	**27.26 ± 0.31**	**19.04 ± 0.03**	**52.58 ± 0.60**	**25.65 ± 0.09**	**18.80 ± 0.03**	**20.33 ± 0.51**	**4.33 ± 0.10**	**2.16 ± 0.05**	**0.60 ± 0.08**
**Mountain region** **Multifloral**	mMR1	14.655 ± 0.064	2.888 ± 0.075	23.29 ± 0.17	8.887 ± 0.041	41.75 ± 1.93	13.96 ± 0.73	10.67 ± 1.27	15.85 ± 0.15	0.902 ± 0.060	0.368 ± 0.021	-
	mMR2	16.460 ± 0.014	2.45 ± 0.00	17.40 ± 0.26	13.71 ± 0.36	41.28 ± 1.43	16.38 ± 0.40	13.60 ± 0.56	8.74 ± 0.30	2.23 ± 0.12	0.327 ± 0.043	-
	mMR3	13.780 ± 0.028	2.08 ± 0.00	17.31 ± 0.22	13.35 ± 0.43	49.42 ± 0.13	18.64 ± 0.38	14.302 ± 0.015	11.95 ± 0.28	3.129 ± 0.057	1.158 ± 0.010	0.242 ± 0.083
	mMR4	22.210 ± 0.085	1.84 ± 0.00	15.59 ± 0.20	14.57 ± 0.14	51.29 ± 0.82	19.88 ± 0.36	18.70 ± 0.50	7.60 ± 0.33	4.98 ± 0.29	-	0.13 ± 0.00
	mMR5	19.780 ± 0.042	2.375 ± 0.011	16.76 ± 0.20	6.13 ± 0.19	43.68 ± 1.04	18.841 ± 0.046	15.36 ± 0.94	5.75 ± 0.13	3.56 ± 0.10	0.010 ± 0.021	0.156 ± 0.022
	mMR6	17.150 ± 0.085	2.25 ± 0.00	18.171 ± 0.043	6.94 ± 0.12	45.07 ± 0.71	18.40 ± 0.27	15.31 ± 0.25	8.41 ± 0.15	2.81 ± 0.39	0.017 ± 0.015	0.122 ± 0.041
	**Mean**	**17.34 ± 0.05**	**2.33 ± 0.01**	**18.09 ± 0.18**	**10.60 ± 0.21**	**45.42 ± 1.01**	**17.68 ± 0.36**	**14.66 ±0.59**	**9.72 ± 0.22**	**2.94 ± 0.17**	**0.38 ± 0.02**	**0.16 ± 0.04**
	**Minimum**	**13.78 ± 0.03**	**1.84 ± 0.00**	**15.59 ± 0.20**	**6.13 ± 0.19**	**40.85 ± 1.93**	**13.96 ± 0.73**	**10.67 ± 1.27**	**5.75 ± 0.13**	**2.23 ± 0.12**	**0.01 ± 0.02**	**0.12 ± 0.04**
	**Maximum**	**22.21 ± 0.09**	**2.89 ± 0.08**	**23.29 ± 0.17**	**14.57 ± 0.14**	**51.29 ± 0.82**	**19.88 ± 0.36**	**18.70 ± 0.50**	**15.85 ± 0.15**	**4.98 ± 0.29**	**1.16 ± 0.01**	**0.24 ± 0.08**
**Mountain region** **Unifloral**	*P. spinosa*	11.295 ± 0.011	3.021 ± 0.016	23.719 ± 0.48	8.534 ± 0.080	42.32 ± 0.10	12.28 ± 0.23	10.61 ± 0.14	18.68 ± 0.43	0.438 ± 0.076	0.315 ± 0.033	-
	*P. avium*	15.525 ± 0.021	3.81 ± 0.00	21.97 ± 0.58	9.10 ± 0.23	43.65 ± 0.82	12.39 ± 0.43	9.82 ± 0.23	17.84 ± 0.26	3.228 ± 0.046	0.206 ± 0.071	0.159 ± 0.017
	*Salix* spp.	16.760 ± 0.014	2.39 ± 0.00	17.35 ± 0.42	12.07 ± 0.19	45.56 ± 0.48	18.63 ± 0.53	16.01 ± 0.52	9.60 ± 0.21	1.17 ± 0.30	0.155 ± 0.025	-
	*T. officinale*	16.175 ± 0.049	1.18 ± 0.00	13.90 ± 0.17	17.531 ± 0.025	42.11 ± 1.12	19.19 ± 0.14	17.20 ± 0.41	3.79 ± 0.62	1.483 ± 0.086	0.36 ± 0.00	0.09 ± 0.13
	*Q. pubescens*	26.475 ± 0.106	2.047 ± 0.029	16.423 ± 0.067	11.55 ± 0.13	51.70 ± 0.11	24.21 ± 0.21	19.856 ± 0.025	4.09 ± 0.13	3.31 ± 0.23	0.05 ± 0.00	0.176 ± 0.028
	*J. regia*	20.515 ± 0.064	2.057 ± 0.019	16.39 ± 0.12	13.73 ± 0.31	49.26 ± 0.45	20.353 ± 0.010	18.286 ± 0.071	8.14 ± 0.36	1.756 ± 0.065	0.638 ± 0.022	0.18 ± 0.00
	*F. vulgaris*	17.680 ± 0.014	2.08 ± 0.00	14.33 ± 0.29	4.51 ± 0.11	45.06 ± 0.97	19.291 ± 0.036	16.02 ± 0.75	7.36 ± 0.00	2.22 ± 0.11	0.106 ± 0.099	0.061 ± 0.011
	*M. sativa*	16.57 ± 0.11	2.823 ± 0.059	23.56 ± 0.24	8.863 ± 0.025	41.64 ± 0.62	18.73 ± 0.12	14.14 ± 0.47	3.00 ± 0.10	5.480 ± 0.012	-	0.289 ± 0.086
	*P. tanacetifolia*	17.645 ± 0.021	2.714 ± 0.010	26.32 ± 0.11	5.847 ± 0.026	50.86 ± 0.69	24.284 ± 0.042	18.49 ± 0.22	4.35 ± 0.54	3.350 ± 0.088	0.300 ± 0.023	0.087 ± 0.066
	**Mean**	**17.63 ± 0.05**	**2.46 ± 0.01**	**19.33 ± 0.28**	**11.76 ± 0.13**	**45.80 ± 0.60**	**18.82 ± 0.19**	**15.60 ± 0.32**	**8.54 ± 0.29**	**2.49 ± 0.11**	**0.27 ± 0.03**	**0.15 ± 0.05**
	**Minimum**	**11.30 ± 0.01**	**1.18 ± 0.00**	**13.90 ± 0.17**	**4.51 ± 0.11**	**41.64 ± 0.62**	**12.28 ± 0.23**	**9.82 ± 0.23**	**3.00 ± 0.10**	**0.44 ± 0.08**	**0.05 ± 0.00**	**0.06 ± 0.01**
	**Maximum**	**26.48 ± 0.11**	**3.81 ± 0.00**	**26.32 ± 0.11**	**17.53 ± 0.03**	**51.70 ± 0.11**	**24.28 ± 0.04**	**19.86 ± 0.03**	**18.68 ± 0.43**	**5.48 ± 0.01**	**0.64 ± 0.02**	**0.29 ± 0.09**
**Adriatic region** **Multifloral**	mAR1	15.005 ± 0.049	2.49 ± 0.00	19.536 ± 0.075	8.883 ± 0.083	35.79 ± 0.31	14.23 ± 0.28	11.809 ± 0.061	8.50 ± 0.54	0.96 ± 0.11	0.286 ± 0.066	0.01 ± 0.00
	mAR2	22.84 ± 0.14	2.080 ± 0.027	17.211 ± 0.092	10.80 ± 0.24	46.66 ± 0.080	22.602 ± 0.019	16.799 ± 0.032	3.17 ± 0.13	3.881 ± 0.077	-	0.212 ± 0.012
	mAR3	14.18 ± 0.23	2.26 ± 0.016	21.190 ± 0.041	8.52 ± 0.11	43.19 ± 0.64	23.48 ± 0.12	14.27 ± 0.48	2.62 ± 0.34	2.730 ± 0.026	-	0.097 ± 0.087
	mAR4	19.31 ±0.12	2.06 ± 0.00	20.02 ± 0.10	9.790 ± 0.053	47.90 ± 0.25	23.88 ± 0.43	16.378 ± 0.018	5.15 ± 0.23	2.270 ± 0.015	0.137 ± 0.089	0.09 ± 0.00
	**Mean**	**17.83 ± 0.13**	**2.22 ± 0.01**	**19.49 ± 0.08**	**9.50 ± 0.12**	**43.39 ± 0.32**	**21.05 ± 0.21**	**14.81 ± 0.15**	**4.86 ± 0.31**	**2.46 ± 0.06**	**0.21 ± 0.08**	**0.10 ± 0.02**
	**Minimum**	**14.18 ± 0.23**	**2.06 ± 0.00**	**17.21 ± 0.09**	**8.52 ± 0.11**	**35.79 ± 0.31**	**14.23 ± 0.28**	**11.81 ± 0.06**	**2.62 ± 0.34**	**0.96 ± 0.11**	**0.14 ± 0.09**	**0.01 ± 0.00**
	**Maximum**	**22.84 ± 0.14**	**2.49 ± 0.00**	**21.19 ± 0.04**	**10.80 ± 0.24**	**47.90 ± 0.25**	**23.88 ± 0.43**	**16.80 ± 0.03**	**8.50 ± 0.54**	**3.88 ± 0.08**	**0.29 ± 0.07**	**0.21 ± 0.01**
**Adriatic region** **Unifloral**	*C. biennis*	17.49 ± 0.17	1.563 ± 0.051	16.60 ± 0.00	14.49 ± 0.25	43.83 ± 1.12	20.08 ± 0.99	15.51 ± 0.80	4.45 ± 0.24	3.38 ± 0.90	0.23 ± 0.00	0.186 ± 0.010
	*P. mahaleb*	12.745 ± 0.04	3.089 ± 0.024	22.23 ± 0.16	8.62 ± 0.13	43.32 ± 2.61	14.17 ± 0.83	11.95 ± 0.67	14.37 ± 0.78	2.55 ± 0.30	0.089 ± 0.032	0.194 ± 0.061
	*Q. pubescens*	16.14 ± 0.26	2.217 ± 0.014	18.12 ± 0.25	10.21 ± 0.11	46.73 ± 1.30	19.26 ± 0.56	14.55 ± 0.45	10.17 ± 0.34	2.23 ± 0.13	0.367 ± 0.021	0.149 ± 0.040
	**Mean**	**15.46 ± 0.16**	**2.29 ± 0.03**	**18.98 ± 0.14**	**11.11 ± 0.16**	**44.63 ± 1.68**	**17.84 ± 0.79**	**14.00 ± 0.64**	**9.66 ± 0.45**	**2.72 ± 0.44**	**0.23 ± 0.02**	**0.18 ± 0.04**
	**Minimum**	**12.75 ± 0.04**	**1.56 ± 0.05**	**16.60 ± 0.00**	**8.62 ± 0.13**	**43.32 ± 2.61**	**14.17 ± 0.83**	**11.95 ± 0.67**	**4.45 ± 0.24**	**2.23 ± 0.13**	**0.09 ± 0.03**	**0.15 ± 0.04**
	**Maximum**	**17.49 ± 0.17**	**3.09 ± 0.02**	**22.23 ± 0.16**	**14.49 ± 0.25**	**46.73 ± 1.30**	**20.08 ± 0.99**	**15.51 ± 0.80**	**14.37 ± 0.78**	**3.38 ± 0.90**	**0.37 ± 0.02**	**0.19 ± 0.06**
	**p**	**0.92**	**0.80**	**0.63**	**0.85**	**0.75**	**0.52**	**0.72**	**0.41**	**0.28**	**0.04**	**0.80**

* physico-chemical data are expressed as mean value ± standard deviation (SD) of two replicate measurements (% of dry weight; an exception is moisture content, which is expressed as % of fresh weight); mCR—multifloral bee pollen sample from continental region; mMR—multifloral bee pollen sample from mountain region; mAR—multifloral bee pollen sample from Adriatic region; p—one way ANOVA effect showing the statistical significance of differences (95% confidence interval) between multifloral and unifloral bee pollen from three climatic-geographical regions.

**Table 3:** Volatile headspace compounds (%) from the bee pollen samples collected from continental region.

No.	Compound		Sample *								
		RI	A	B	C	D	E	F	G	H	I
1.	Butanal	<900	-	-	-	-	-	-	-	20.96	5.54
2.	3-Methylbutanal	<900	-	-	-	3.29	-	-	3.38	8.85	2.54
3.	2-Methylbutanal	<900	-	-	-	-	-	-	4.59	-	2.43
4.	3-Hydroxybutan-2-one (acetoin)	<900	-	-	-	-	5.06	-	-	-	-
5.	3-Methylbutan-1-ol	<900	-	-	2.54	-	-	-	1.46	-	0.65
6.	Dimethyl disulfide	<900	-	-	-	2.49	-	-	-	-	-
7.	Pentanal	<900	0.18	-	-	-	4.21	-	2.08	4.74	0.79
8.	3-Methylpentanal **	<900	-	-	-	-	-	-	-	-	0.34
9.	Butanoic acid	<900	3.33	4.51	-	-	-	-	-	-	-
10.	Butan-1,3-diol	<900	-	-	-	2.72	-	-	-	-	-
11.	Butan-2,3-diol	<900	-	-	-	3.37	-	-	-	-	-
12.	Hexanal	<900	0.84	1.14	1.64	3.20	4.59	7.34	2.11	5.40	1.55
13.	(*E*)-Hex-2-enal	<900	-	-	-	-	-	-	-	-	-
14.	Hexan-1-ol	<900	-	-	4.95	-	-	-	-	-	-
15.	Cyclopent-2-ene-1,4-dione	<900	1.06	2.23	-	6.14	-	-	-	-	-
16.	Heptanal	904	0.75	1.97	-	2.12	-	-	1.21	-	0.78
17.	Methyl allyl disulfide	925	-	-	-	3.07	-	-	-	-	-
18.	Methyl hexanoate	928	-	-	-	-	1.90	-	-	-	-
19.	α-Pinene	942	-	-	1.85	-	-	-	-	-	-
20.	Methyl (*Z*)-prop-1-enyl disulfide	948	-	-	-	1.02	-	-	-	-	-
21.	γ-Valerolactone	960	1.59	1.61	-	-	-	-	-	-	-
22.	Benzaldehyde	969	1.09	1.61	-	29.11	1.44	-	40.34	18.68	27.46
23.	Dimethyl trisulfide	979	-	-	-	3.83	-	-	-	-	-
24.	β-Pinene	985	-	-	1.69	-	-	-	-	-	-
25.	Hexanoic acid	986	1.20	3.15	-	-	24.46	-	-	-	-
26.	6-Methylhept-5-en-2-one	989	12.39	21.45	-	3.16	-	8.61	0.51	3.07	-
27.	Octanal	1005	-	-	-	-	-	-	-	1.46	-
28.	δ-3-Carene	1016	-	-	6.72	-	-	-	-	-	-
29.	(*E*,*E*)-Hepta-2,4-dienal	1015	-	-	-	-	-	-	-	-	-
30.	*p*-Cymene	1031	-	-	1.85	-	-	-	-	-	-
31.	2-Ethylhexan-1-ol	1035	-	-	-	-	-	-	1.16	6.15	1.38
32.	γ-Terpinene	1036	-	-	7.02	-	-	-			
33.	Benzyl alcohol	1046	0.23	-	-	1.21	-	-	-	-	3.28
34.	Phenylacetaldehyde	1050	-	-	-	-	-	-	1.16	-	3.07
35.	(*E*)-β-Ocymene	1053	-	-	-	-	-	5.04	-	-	-
36.	(*E*,*E*)-Octa-3,5-dien-2-one	1075	-	-	-	0.41	2.55	-	-	4.74	-
37.	Diallyl disulfide	1084	-	-	-	2.77	-	-	-	-	-
38.	Nonan-2-one	1093	-	-	-	-	-	-	-	-	-
39.	(*E*,*Z*)-Octa-3,5-dien-2-one	1096	-	-	-	0.30	-	-	-	2.68	-
40.	Undecane	1100	-	-	2.03	-	-	-	0.53	1.43	-
41.	Linalool	1103	-	-	-	-	-	-	2.25	-	4.06
42.	Nonanal	1105	8.92	15.22	46.70	14.38	8.37	59.76	6.98	7.84	7.53
43.	2-Phenylethanol	1122	-	-	-	-	-	-	1.35	-	1.68
44.	Methyl octanoate	1128	-	-	-	-	4.46	-	-	-	-
45.	Lilac aldehyde A	1147	-	-	-	-	-	-	2.57	-	-
46.	Phenylacetonitrile	1148	-	-	-	-	-	-	-	-	0.44
47.	Lilac aldehyde B	1155	-	-	-	-	-	-	2.94	-	-
48.	Lilac aldehyde D	1169	-	-	-	-	-	-	1.27	-	-
49.	Octanoic acid	1186	-	-	-	-	8.78	-	-	-	-
50.	Decanal	1207	-	0.92	7.74	-	-	-	-	-	-
51.	Verbenone	1211	-	-	-	-	-	-	13.11	-	21.44
52.	4-Methoxybenzaldehyde	1261	-	-	-	0.07	-	-	-	-	-
53.	Tetradecane	1400	0.48	1.05	-	-	-	-	-	-	0.83
54.	1,3,5-Trimethoxybenzene	1411	0.62	1.30	-	1.71	-	-	-	-	-
55.	(*E*)-Geranyl acetone	1455	5.46	16.19	-	-	-	-	-	-	-
56.	Pentadecane	1500	-	9.76	-	9.63	-	-	-	-	-
57.	β-Tumerone	1668	-	0.87	-	-	-	-	-	-	-
58.	Heptadecane	1700	0.62	1.41	-	-	-	-	-	-	-
59.	Nonadecane	1900	1.66	3.17	-	-	-	3.26	-	-	-
60.	(*E*,*E*)-Geranyl linalool	2038	49.27	-	-	-	-	-	-	-	-
61.	Heneicosane	2100	2.14	5.42	2.28	-	20.17	-	-	-	-
62.	Docosane	2200	0.17	-	-	-	-	-	-	-	-

* A, *R. acris*; B, *F. excelsior;* C, *P. sylvestris;* D, *A. schoenoprasum*; E, *C. biennis*; F, *J. regia*; G, *Quercus* spp.; H, *A. hippocastanum;* I, *Salix* sp.; ** the compound is tentatively identified; RI, retention indices on HP-5MS column.

**Table 4:** Volatile headspace compounds (%) from the bee pollen samples collected from mountain region.

No.	Compound		Sample *					
		RI	A	B	C	D	E	F
1.	Acetic acid	<900	-	-	-	10.10	21.34	21.69
2.	Ethanol	<900	-	-	-	6.93	7.17	9.44
3.	Butanal	<900	-	-	4.66	-	-	-
4.	3-Methylbutanal	<900	-	3.73	4.57	-	-	-
5.	2-Methylbutanal	<900	-	3.71	5.42	-	-	-
6.	3-Hydroxybutan-2-one (acetoin)	<900	-	5.47	3.05	-	11.87	7.07
7.	3-Methylbutan-1-ol	<900	-	3.08	2.84	0.29	-	-
8.	Pentanal	<900	-	4.14	-	-	-	11.85
9.	Butane-1,3-diol	<900	-	-	-	-	1.52	-
10.	Butane-2,3-diol	<900	-	-	-	-	1.80	-
11.	(*E*)-Hex-2-enal	<900	-	-	-	0.12	-	0.94
12.	3-Methylpentanal **	<900	-	-	10.37	-	-	-
13.	Hexanal	<900	0.40	7.02	1.62	1.22	2.89	-
14.	Hexan-1-ol	<900	-	-	-	1.46	1.62	2.63
15.	Heptanal	904	-	5.17	-	0.49	-	5.37
16.	γ-Butyrolactone	922	0.09	-	-	0.43	0.66	-
17.	Methyl hexanoate	928	-	-	0.53	-	0.48	0.60
18.	α-Pinene	942	-	-	-	0.45	-	-
19.	3-Methylpentanoic acid	954	-	-	3.12	-	-	-
20.	γ-Valerolactone	960	-	-	-	0.37	-	-
21.	Benzaldehyde	969	0.65	11.93	28.98	0.63	2.73	1.39
22.	β-Pinene	985	-	-	-	0.94	0.89	-
23.	6-Methylhept-5-en-2-one	989	0.25	2.68	2.20	0.80	2.05	2.96
24.	β-Myrcene	993	-	-	-	1.46	-	-
25.	Octanal	1005	0.14	1.90	0.48	0.55	0.91	1.35
26.	(*E*,*E*)-Hepta-2,4-dienal	1015	-	-	-	0.34	0.39	2.09
27.	β-Phellandrene	1034	-	-	-	5.69	-	-
28.	2-Ethylhexan-1-ol	1035	-	-	-	0.11	-	-
29.	1,8-Cineole	1039	-	-	-	-	0.77	-
30.	Benzyl alcohol	1046	-	-	1.17	-	-	-
31.	Phenylacetaldehyde	1050	1.04	0.38	3.95	0.25	2.28	1.35
32.	(*E*)-β-Ocymene	1053	-	0.92	-	-	-	-
33.	(*E*,*E*)-Octa-3,5-diene-2-one	1075	-	-	0.54	2.46	3.52	5.08
34.	(*E*,Z)-Octa-3,5-diene-2-one	1096	-	-	-	0.45	0.44	1.85
35.	Linalool	1103	-	-	-	-	0.91	-
36.	Nonanal	1105	1.53	9.28	1.24	8.09	3.81	15.57
37.	Methyl octanoate	1128	-	-	0.89	-	1.87	0.38
38.	Lilac aldehyde A	1147	14.84	6.06	3.85	-	-	-
39.	Lilac aldehyde B	1155	29.30	10.43	6.42	-	-	-
40.	Lilac aldehyde D	1169	13.37	3.94	2.48	-	-	-
41.	Ethyl octanoate	1198	-	-	-	-	0.48	-
42.	Dodecane	1200	-	-	0.66	-	0.80	-
43.	Lilac alcohol A	1205	11.37	0.24	-	-	-	-
44.	Decanal	1207	-	-	-	1.11	-	-
45.	Verbenone	1211	-	-	-	-	1.22	-
46.	Lilac alcohol B	1214	13.76	0.59	-	-	-	-
47.	β-Cyclocitral	1223	1.90	-	-	-	-	-
48.	Lilac alcohol D	1232	4.21	-	-	-	-	-
49.	Bornyl acetate	1287	-	-	-	0.82	-	-
50.	Tetradecane	1400	-	-	-	0.61	0.36	0.39
51.	α-Ionone	1429	0.53	-	-	-	-	-
52.	Pentadecane	1500	-	10.29	-	51.16	-	-
53.	(*E*)-β-Ionone	1512	1.43	-	-	-	-	-
54.	Heneicosane	2100	-	-	-	-	18.23	-

* A, *P. avium*; B, *Salix* spp.; C, *T. officinale*; D, *P. sylvestris*; E, *C. biennis*; F, *Q. pubescens*; ** the compound is tentatively identified; RI, retention indices on HP-5MS column.

**Table 5:** Volatile headspace compounds (%) from the bee pollen samples collected from Adriatic region.

No.	Compound		Sample *					
		RI *	A	B	C	D	E	F
1.	Acetaldehyde	<900	5.63	-	-	-	-	-
2.	Ethanol	<900	4.39	3.01	3.14	2.99	-	-
3.	Dimetyl sulfide	<900	-	4.30	-	6.48	-	-
4.	Acetic acid	<900	6.93	9.30	9.27	19.09	-	-
5.	3-Methylbutanal	<900	2.87	1.86	-	-	-	-
6.	3-Hydroxy-butan-2-one (acetoin)	<900	5.05	3.99	2.68	3.67	19.41	-
7.	Pentanal	<900	-	1.46	1.24	-	5.83	-
8.	Butane-1,3-diol	<900	0.53	1.36	0.27	1.17	-	-
9.	Butane-2,3-diol	<900	0.62	1.23	0.43	1.12	-	-
10.	Hexanal	<900	4.88	3.56	2.54	2.45	5.22	1.44
11.	4-Methylpentan-1-ol	<900	-	-	2.75	-	-	-
12.	(*E*)-Hex-2-enal	<900	0.30	-	0.24	0.52	-	-
13.	Hexan-1-ol	<900	-	0.67	0.15	-	-	-
14.	Heptan-2-one	<900	-	-	2.08	-	-	-
15.	Heptan-2-ol	902	-	-	30.63	-	-	-
16.	Heptanal	904	4.46	2.19	-	1.50	-	-
17.	γ-Butyrolactone	922	-	-	2.13	0.34	0.93	0.30
18.	Methyl hexanoate	928	-	-	-	0.45	0.70	-
19.	α-Pinene	942	-	0.39	-	0.19	-	-
20.	Benzaldehyde	969	4.88	6.36	3.62	5.60	3.14	2.09
21.	Sabinene	982	-	-	-	-	1.21	-
22.	β-Pinene	985	2.16	2.37	0.86	1.57	-	-
23.	Hexanoic acid	986	-	-	-	-	-	-
24.	6-Methylhept-5-en-2-one	989	5.81	8.33	1.91	9.56	2.50	1.53
25.	β-Myrcene	993	-	-	-	-	-	-
26.	Octanal	1005	-	0.89	0.33	-	-	0.57
27.	(*E*,*E*)-Hepta-2,4-dienal	1015	-	-	0.65	-	-	
28.	2-Ethylhexan-1-ol	1035	-	-	-	1.23	5.41	0.87
29.	1,8-Cineole	1039	-	0.99	-	2.45	-	-
30.	Benzyl alcohol	1046	-	1.04	-	3.26	-	-
31.	Phenylacetaldehyde	1050	5.46	5.69	3.67	11.79	1.57	0.44
32.	(*E*)-β-Ocymene	1053	4.06	5.27	-	-	-	
33.	(*E*,*E*)-Octa-3,5-diene-2-one	1075	0.42	2.40	5.03	1.77	1.82	0.33
34.	(*E*,*Z*)-Octa-3,5-diene-2-one	1096	0.30	0.29	-	-	-	-
35.	Undecane	1100	-	-	-	-	0.98	0.54
36.	Linalool	1103	1.79	1.94	0.45	0.87	-	-
37.	Nonanal	1105	28.67	15.53	4.60	8.14	3.65	5.19
38.	β-Thujone	1109	-	-	-	1.21	-	-
39.	2-Phenylethanol	1122	-	2.60	-	0.91	-	-
40.	Methyl octanoate	1128	-	0.99	-	0.64	4.35	-
41.	Lilac aldehyde A	1146	-	-	3.12	-	5.61	14.69
42.	Lilac aldehyde B	1155	-	-	5.20	-	9.00	25.04
43.	Lilac aldehyde D	1169	-	-	2.16	-	3.28	14.51
44.	Ethyl octanoate	1198	-	-	-	-	1.68	-
45.	Dodecane	1200	-	0.51	0.39	0.40	0.62	-
46.	Lilac alcohol A	1205	-	-	-	-	-	2.99
47.	Decanal	1207	1.35	0.77	-	-	-	-
48.	Verbenone	1211	-	-	-	-	1.09	-
49.	Lilac alcohol B	1214	-	-	0.24	-	-	5.32
50.	β-Cyclocitral	1223	-	-	-	-	-	3.28
51.	Lilac alcohol D	1232	-	-	-	-	-	1.10
52.	Nerol	1234	-	-	1.22	-	-	-
53.	4-Methoxybenzaldehyde	1264	-	-	-	-	-	5.66
54.	Methyl decanoate	1327	-	0.49	-	-	0.81	-
55.	Tetradecane	1400	-	-	-	0.96	-	0.27
56.	α-Ionone	1429	-	-	-	-	-	0.92
57.	(*E*)-Geranyl acetone	1455	2.44	3.51	-	4.07	-	-
58.	(*E*)-β-Ionone	1512	-	-	-	-	-	2.24
59.	β-Tumerone	1668	-	-	-	0.69	-	-
60.	Heneicosane	2100	-	1.74	-	0.90	6.17	0.66

* A, *pubescens*; B, *Malus* spp.; C, *S. vulgaris*; D, *R. lutea*; E, *C. biennis*; F, *P. mahaleb*; RI, retention indices on HP-5MS column.

## Data Availability

Not applicable.

## Supplementary Materials

The following are available online at https://www.mdpi.com/article/10.3390/foods10092103/s1, Supplementary Materials S1: The effects of classical ANOVA and Kruskal–Wallis one-way ANOVA showing differences between multifloral and unifloral bee pollen originating from different regions for 11 physico-chemical measurement data (moisture, ash, proteins, total lipids, total sugars, fructose, glucose, sucrose, maltose, melezitose, and raffinose content), Supplementary Materials S2: Descriptive sheets for unifloral bee pollen collected from three Croatian regions (continental-CR, mountain-MR, Adriatic-AR): visual identity (color), nutritional value (content of ash, proteins, total lipids, total sugars, fructose, glucose and sucrose; expressed as a percentage of the total weight), and FTIR-ATR spectra (fingerprint region: 1800–650 cm^−1^).
